# Incidence and Mortality of Sarcomas in Shanghai, China, During 2002–2014

**DOI:** 10.3389/fonc.2019.00662

**Published:** 2019-07-17

**Authors:** Bao Pingping, Zhou Yuhong, Lu Weiqi, Wu Chunxiao, Wang Chunfang, Sun Yuanjue, Zhang Chenping, Xiao Jianru, Lu Jiade, Kong Lin, Cai Zhengdong, Zhang Weibin, Fu Chen, Yao Yang

**Affiliations:** ^1^Department of Cancer Control and Prevention, Shanghai Municipal Center for Disease Prevention and Control, Shanghai, China; ^2^Department of Medical Oncology, Zhongshan Hospital, Fudan University, Shanghai, China; ^3^Department of General Surgery, Zhongshan Hospital, Fudan University, Shanghai, China; ^4^Department of Medical Oncology, Sixth People's Hospital South Campus, Shanghai Jiaotong University School of Medicine, Shanghai, China; ^5^Department of Oral and Maxillofacial Surgery, Shanghai Ninth People's Hospital, Shanghai Jiaotong University School of Medicine, Shanghai, China; ^6^Department of Bone Tumor Surgery, Shanghai Changzheng Hospital, Shanghai, China; ^7^Department of Radiation Oncology, Shanghai Proton and Heavy Ion Center, Shanghai Cancer Hospital, Fudan University, Shanghai, China; ^8^Department of Orthopedics, Shanghai General Hospital, Shanghai Jiaotong University School of Medicine, Shanghai, China; ^9^Department of Orthopedics, Shanghai Ruijin Hospital, Shanghai Jiaotong University School of Medicine, Shanghai, China; ^10^Shanghai Municipal Center for Disease Prevention and Control, Shanghai, China; ^11^Department of Medical Oncology, Shanghai Jiao Tong University Affiliated Sixth People's Hospital, Shanghai, China

**Keywords:** sarcoma, incidence, mortality, epidemiology, population-based cancer registry

## Abstract

**Background:** Sarcomas are a heterogeneous group of rare but deadly malignant tumors. The aim of this study was to comprehensively describe the incidence and mortality of sarcomas in Shanghai during 2002–2014.

**Method:** Data were from Shanghai Cancer Registry. All new cases diagnosed with sarcomas and all death records where the cause of death listed as sarcomas were included. The characteristics of sarcomas incidence and mortality were analyzed. Age-standardized rates (ASRs) were adjusted by the world standard population. The trends were assessed by Joinpoint analysis.

**Results:** A total of 9,440 incident cases were identified. The ASR was 3.4/10^5^ for all sarcomas combined. Incidence of sarcomas overall was similar in females (3.5/10^5^) as in males (3.4/10^5^). Except for sarcomas “Not Otherwise Specified” (NOS), the most common histological subtype was gastrointestinal stromal sarcoma (GISS) (14.8%), which was followed by fibrosarcoma (7.2%), lipoblastoma (6.7%), leiomyosarcomas (6.5%), and osteosarcoma (5.3%). Among those incident cases, 87.9% were located in soft tissue sarcomas (STS) and 12.1% in bone and joint (bone sarcomas). The ASRs for STS and bone sarcomas were 2.8/10^5^ and 0.6/10^5^, respectively. Incidence rates for all STS combined rose exponentially with age, while bone sarcomas had the highest incidence at age 0–19.

There were 4,279 deaths during 2002–2014 with the ASR of 1.3/10^5^. Age-adjusted mortality due to sarcomas was slightly higher in males (1.5/10^5^) than females (1.2/10^5^). Except for sarcomas NOS, leiomyosarcomas was the most common subtype, comprising 9.9% of deaths due to sarcomas, followed by lipoblastoma (6.4%) and osteosarcoma (6.3%). The ASRs of mortality for STS and bone sarcomas were 1.0/10^5^ and 0.2/10^5^, respectively.

For both males and females, the age-standardized incidence for STS and bone sarcomas did not change meaningfully over the study period. In contrast, age-standardized STS mortality in females increased by 2.3% per year (95% CI: 0.3, 4.4%), but was unchanged in males. No meaningful trends in bone sarcomas mortality were observed for either males or females.

**Conclusion:** This population-based study was the first report of epidemiology of sarcomas in Shanghai according to anatomic site and histologic type. The diversity and rarity of sarcomas suggested more detailed data are warranted.

## Introduction

Sarcomas, a heterogeneous group of rare malignant tumors arising from mesenchymal cells, account for about 1% of all new malignancies diagnosed (1, 2). These tumors can occur at any age and in almost any anatomic site. In relation to the anatomy, there have two types of common and distinct sarcomas: sarcomas from bone and joint (bone sarcomas) and soft tissue sarcomas (STS). Based on the histology, more than 50 distinct histological sarcoma subtypes exist according to the classification of the World Health Organization (WHO) updated in 2002 (3). It's difficult to obtain the precise estimates of sarcomas and sarcoma subtypes. The patterns of incidence and mortality of sarcomas have little been studied (1, 4, 5). Sarcomas, although relatively rare, are quite deadly and disproportionately affect younger population. STS are reported to account for, respectively, 0.7–1% and 4–8% of all adult and pediatric malignant tumors, and bone sarcomas for, respectively, 0.2% and 5% in most comprehensive reviews (1, 6–8).

Sarcomas can originate from any organ, tissue, bone, or cartilage. STS diagnoses predominate over bone sarcoma diagnoses with about 4:1 incidence ratio (5). A study on sarcomas of all types combined from RARECARE project showed that 84% were STS and 14% were bone sarcomas, of which age-standardized incidence of STS was 4.2/10^5^ and that of bone sarcomas was 0.8/10^5^ in Europe (4). In Surveillance, Epidemiology, and End Results (SEER) program data, STS also occurred much more frequently than bone sarcomas, which accounted for nearly 87% and 13% in 2008, respectively (1). No population-based mortality data have been reported before.

The causes of most sarcomas are unknown. Environmental factors, including ionizing radiation, occupational exposure to certain chemicals such as herbicide, have been associated with increased risk of specific types of sarcomas. Several heritable syndromes are associated with the development of some sarcomas [e.g., heritable retinoblastoma, neurofibromatosis 1, Li-Fraumeni syndrome (LFS)] (5, 9).

The aim of this paper is to examine incidence, mortality, and the temporal trends for sarcomas in Shanghai from 2002 through 2014, based on a population-based cancer registry, according to anatomic site and histologic type/subtype, using the most recent criteria of the WHO classification (10). These population-based data will be important in furthering our understanding of the morphologic and genetic diversity of sarcomas.

## Methods

Population-based cancer incidence and mortality data were derived from the Shanghai Cancer Registry (SCR), a member of the International Association of Cancer Registries (IACR). SCR has been a regular contributor to the *Cancer Incidence in Five Continents* (*CI5*) published by the IARC and the data have been published in the last seven volumes of *CI5*. Details of the cancer registry have been previously described (11, 12). Briefly, the SCR has formed standard system to collect, process, and report cancer incidence data. A standardized notification card, which includes information on name, date of birth, gender, address, occupation, primary site of cancer, histopathology, incidence date, basis of cancer diagnosis, and reporting hospital is used for reporting cancer cases. Death certificates have been used to gather information on unregistered cancer patient and all death cases due to sarcoma based entirely on vital statistics records during the time period have been included in SCR. The data for incidence and mortality of sarcomas during this period were complete in this study. The completeness of coverage of the Registry is very high with death certification only (DCO) <1%. Sarcomas cases (including second primary cancers with 0.5%) from soft tissues or bone, diagnosed among residents of Shanghai during 2002–2014, were included in this study.

Primary site and histological type were coded according to the third edition of the International Classification of Disease for Oncology (ICD-O-3) and then categorized into major histological types and subtypes of sarcoma as shown in Table 1. In brief, the cancers described in this manuscript include all sarcomas from soft tissue and from bone, including ICD-O-3 M codes 8800-8935, 8910, 8920, 8936, 8940, 8950-8959, 8963-8964, 8990-8991, 9020-9044, 9120-9133, 9150, 9170, 9180-9251, 9260-9261, 9364-9372, 9540-9581 combined with all ICD-O-3 T codes (C00-C80). There were 546 cases from C49 with M 8000-8004 included in this study. Finally, a total 9,440 incident cases and 4,279 death cases that met these criteria were included in the study. The percentage of histologically verified cases (MV%) was 94.4% and the death certificate only (DCO) % was 0.15%.

**Table 1:** Histological group of sarcomas by ICD-O-3 code.

**Histological group**	**ICD-O-3 codes**
Sarcoma NOS	M8800-8806, M8000-8004 located in C49
Osteosarcoma	M9180-9195
Chondrosarcoma	M9220-9243
Ewing's sarcoma & PNET	M9260, 9261, 9364, 9471, 9473, 9474
Giant cell sarcoma	M9250-9252
Lipoblastoma	M8850-8858
Fibrosarcoma	M8810-8815
Malignant fibrohistiocytoma	M8830
Dermatofibrosarcoma protuberans	M8832,8833
Vascular sarcoma	M8710, 9120-9133, 9150, 9170
Rhabdosarcoma	M8900-8920
leiomyosarcomas	M8890-8896
Gastrointestinal stromal sarcoma	M8936
Ameloblastoma	M9270, 9290, 9310, 9330
Malignant peripheral nerve sheath tumor (MPNST)	M9540-9571
Synovial sarcoma	M9040-9043
Stromal sarcoma	M8930-8935
Clear cell sarcoma	M8964,9044
Myxosarcoma	M8840
Malignant mesenchymoma	M8990
Embryonic sarcoma	M8991
Kaposi's sarcoma	M9140
Granulosa cell sarcoma	M9580
Alveolar soft part sarcoma (ASPS)	M9581

*NOS, not otherwise specified; PNET, primitive neuroectodermal tumors*.

The corresponding population data of Shanghai urban areas were retrieved from the Shanghai Municipal Bureau of Public Security every year. Between 2002 and 2014, Shanghai had a total population of inhabitants 179,955,231. The study was approved and the need for consent was waived by the institutional review board (IRB) of Shanghai Municipal Center for Disease Control and Prevention. In this study, only data in annual cancer report was used and no information to identify individual subjects was included.

The relative frequency was calculated as the percentage contribution of each particular group or subgroup to the total case series. The incidence rate was the number of new cases divided by the population at risk and was expressed as the number per 100,000 at risk and was age-adjusted by the direct method using the weight of the 1960 world standard population (13). The annual percent changes (APCs), representing the average percent increase or decrease in cancer rates per year over a specified period of time, were obtained using the joinpoint regression analysis. The joinpoint analysis has been widely applied to detect the changes points (joinpoints) and determine the trends between join points, which involves fitting a series of joined straight lines on a logarithmic scale to the trends in annual age-standardized rates (ASRs) (14). The allowed maximum number of joinpoints was one over 13 years as at least 5 years was required for each segment. We used a Joinpoint regression model implemented in the Joinpoint Regression Program (Version 4.5.0.1), which was developed by the Surveillance, Epidemiology, and End Results Program of the US National Cancer Institute (15).

## Results

### Incidence

During 2002–2014, 4,503 (47.7%) males and 4,937 (52.3%) females were diagnosed with sarcomas. The crude annual incidence rate (CR) was 5.3/10^5^ and the ASR was 3.4/10^5^. Incidence of sarcomas overall was similar in females (3.5/10^5^) as in males (3.4/10^5^). The number of cases, percent distribution, age distribution, and incidence rates according to the histological group were shown in Table 2. About 3.9% of sarcomas occurred in children and adolescents (0–19 years), while majority (60.9%) occurred in the 20–64 years age group. The remaining 35.2% occurred in the elderly aged over 65 years. The most common histological subtype was gastrointestinal stromal sarcoma (GISS, malignant GISTs, 14.8%), which was followed by fibrosarcoma (7.2%), lipoblastoma (6.7%), leiomyosarcoma (6.5%), and osteosarcoma (5.3%). It should be noted that 31.2% of total cases were sarcomas “Not Otherwise Specified” (NOS). Kaposi sarcoma was very rare in Shanghai with only 14 cases during this whole period. In females, the incidence of stromal sarcoma (ASR 0.2/10^5^), the fifth common subtype, was apparently higher than in males (ASR 0.02/10^5^).

**Table 2:** Incidence of sarcomas by age, gender, and histologic type, Shanghai, 2002–2014.

**Histologic group**	**Male**								**Female**							
			**Incidence rates (1/100,000)**								**Incidence rates (1/100,000)**					
	***N***	**%**	**Ages 0–19**	**Ages 20–44**	**Ages 45–64**	**Ages 65+**	**CR**	ASR^*^	***N***	**%**	**Ages 0–19**	**Ages 20–44**	**Ages 45–64**	**Ages 65+**	**CR**	ASR^*^
Sarcoma NOS	1,390	30.8	0.13	0.49	1.8	4.9	1.5	0.88	1,552	31.4	0.15	0.64	2.1	4.4	1.7	0.96
Osteosarcoma	259	5.8	0.58	0.27	0.22	0.23	0.29	0.34	239	4.8	0.41	0.22	0.25	0.28	0.27	0.29
Chondrosarcoma	91	2.0	0.08	0.06	0.13	0.13	0.10	0.08	102	2.1	0.08	0.07	0.16	0.14	0.11	0.09
Ewing's sarcoma and PNET	48	1.1	0.12	0.05	0.04	0.02	0.05	0.08	41	0.83	0.11	0.05	0.03	0.01	0.05	0.07
Giant cell sarcoma	111	2.5	0.06	0.17	0.12	0.06	0.12	0.11	109	2.2	0.06	0.17	0.13	0.06	0.12	0.10
Lipoblastoma	360	8.0	0.01	0.14	0.56	1.1	0.40	0.23	274	5.6	0.02	0.14	0.44	0.58	0.30	0.17
Fibrosarcoma	364	8.1	0.09	0.28	0.48	0.86	0.40	0.27	314	6.4	0.04	0.25	0.45	0.60	0.35	0.23
Malignant fibrohistiocytoma	201	4.5	0.02	0.07	0.26	0.72	0.22	0.13	161	3.3	0.02	0.08	0.19	0.48	0.18	0.10
Dermatofibrosarcoma protuberans	251	5.6	0.09	0.32	0.34	0.20	0.28	0.21	154	3.1	0.08	0.22	0.17	0.14	0.17	0.14
Vascular sarcoma	98	2.2	0.02	0.02	0.12	0.39	0.11	0.06	94	1.9	0.02	0.05	0.13	0.23	0.10	0.06
Rhabdosarcoma	91	2.0	0.33	0.03	0.08	0.11	0.10	0.18	62	1.3	0.18	0.05	0.04	0.08	0.07	0.11
Leiomyosarcomas	168	3.7	0.00	0.06	0.24	0.56	0.19	0.10	441	8.9	0.02	0.25	0.77	0.79	0.49	0.28
Gastrointestinal stromal sarcoma	663	14.7	0.00	0.13	0.97	2.40	0.74	0.39	732	14.8	0.00	0.13	1.2	2.1	0.81	0.40
Ameloblastoma	4	0.09	0.00	0.00	0.01	0.00	0.00	0.00	4	0.08	0.00	0.00	0.00	0.01	0.00	0.00
Malignant peripheral nerve sheath tumor (MPNST)	217	4.8	0.01	0.16	0.30	0.51	0.24	0.15	221	4.5	0.06	0.17	0.34	0.35	0.25	0.17
Synovial sarcoma	44	0.98	0.04	0.05	0.06	0.04	0.05	0.05	43	0.87	0.01	0.05	0.06	0.05	0.05	0.04
Stromal sarcoma	30	0.67	0.00	0.01	0.04	0.12	0.03	0.02	274	5.6	0.02	0.24	0.46	0.35	0.30	0.20
Clear cell sarcoma	12	0.27	0.01	0.02	0.01	0.02	0.01	0.01	18	0.36	0.01	0.01	0.02	0.04	0.02	0.02
Myxosarcoma	22	0.49	0.00	0.01	0.03	0.09	0.02	0.01	27	0.55	0.00	0.02	0.05	0.03	0.03	0.02
Malignant mesenchymoma	57	1.3	0.01	0.02	0.08	0.18	0.06	0.04	54	1.1	0.01	0.03	0.09	0.11	0.06	0.03
Embryonic sarcoma	1	0.02	0.01	0.00	0.00	0.00	0.00	0.00	3	0.06	0.02	0.00	0.00	0.00	0.00	0.01
Kaposi's sarcoma	10	0.22	0.00	0.01	0.01	0.04	0.01	0.01	4	0.08	0.00	0.00	0.01	0.01	0.00	0.00
Granulosa cell sarcoma	5	0.11	0.00	0.00	0.01	0.02	0.01	0.00	8	0.16	0.00	0.01	0.02	0.00	0.01	0.01
Alveolar soft part sarcoma (ASPS)	6	0.13	0.02	0.01	0.00	0.00	0.01	0.01	6	0.12	0.01	0.02	0.00	0.00	0.01	0.01
Total	4,503	100.0	1.6	2.4	5.9	12.6	5.0	3.4	4,937	100.0	1.3	2.9	7.1	10.9	5.5	3.5

* *Adjusted by the world standard population. CR, crude rate; ASR, age-standardized rate; NOS, not otherwise specified; PNET, primitive neuroectodermal tumors*.

Incidence rates of sarcomas overall increased with age following a modest peak in adolescents. Incidence rates for all STS combined rose exponentially with age, while bone sarcomas had the highest incidence at age 0–19. Among the histological categories, osteosarcoma was the most frequent at age 0–19, with the peak of incidence of 0.5/10^5^. Rhabdosarcoma was the second frequent at this age group. Liposarcoma rates were very low in children and adolescents and then rose exponentially before peaking in the elderly. GISS incidence cases were rare before 40 years old and had the highest incidence at age 65+ group. Leiomyosarcoma incidence rates in females had two peaks at age group of 45–49 and 75–79, respectively.

As Table 3 shown, among those newly diagnosed cases, 87.9% were located in STS and 12.1% in bone and joint (bone sarcomas).The CR and ASR were 4.6/10^5^ and 2.8/10^5^ for STS, 0.6/10^5^ and 0.6/10^5^ for bone sarcomas, respectively. About one-third (32.2%) of STS were located in the connective, subcutaneous and other soft tissues (ICD-10: C49), followed by digestive organs (31.4%), and female genital organs (8.6%).

**Table 3:** Incidence of sarcomas by age, gender, and primary site, Shanghai, 2002–2014.

**ICD-O**	**Primary sites**	**Male**								**Female**							
				**Incidence rates (1/100,000)**								**Incidence rates (1/100,000)**					
		***N***	**%**	**Ages 0–19**	**Ages 20–44**	**Ages 45–64**	**Ages 65+**	**CR**	**ASR**	***N***	**%**	**Ages 0–19**	**Ages 20–44**	**Ages 45–64**	**Ages 65+**	**CR**	**ASR**
C00-14	Lip, oral cavity, and pharynx	40	0.89	0.02	0.03	0.06	0.09	0.04	0.03	15	0.30	0.01	0.01	0.02	0.04	0.02	0.01
C15-26	Digestive organs	1,280	28.4	0.00	0.31	1.9	4.5	1.4	0.76	1,361	27.6	0.04	0.30	2.0	4.1	1.5	0.77
C16	Stomach	669	14.9	0.00	0.13	0.93	2.56	0.74	0.39	798	16.2	0.01	0.14	1.2	2.6	0.89	0.44
C30-39	Respiratory system and intrathoracic organs	210	4.7	0.05	0.06	0.32	0.64	0.23	0.14	119	2.4	0.04	0.10	0.18	0.17	0.13	0.09
C40-41	Bone and Joint	582	12.9	0.77	0.61	0.65	0.62	0.65	0.63	558	11.3	0.61	0.57	0.65	0.66	0.62	0.57
C44	Skin	274	6.1	0.09	0.34	0.34	0.32	0.30	0.23	178	3.6	0.10	0.23	0.19	0.24	0.20	0.16
C47	Peripheral nerve and autonomic nerve system	159	3.5	0.02	0.13	0.21	0.36	0.18	0.12	157	3.2	0.06	0.12	0.24	0.26	0.17	0.12
C48	Retropheritoneum and peritoneum	233	5.2	0.00	0.07	0.36	0.74	0.26	0.14	308	6.2	0.00	0.15	0.52	0.65	0.34	0.19
C49	Connective, subcutaneous, and other soft tissues	1,480	32.9	0.47	0.74	1.8	4.6	1.6	1.1	1,227	24.9	0.30	0.69	1.5	3.3	1.4	0.83
C50	Breast	7	0.16	0.00	0.00	0.01	0.03	0.01	0.00	116	2.4	0.01	0.10	0.22	0.10	0.13	0.08
C51-57	Female genital organs	–	–	–	–	–	–	–	–	718	14.5	0.05	0.49	1.3	0.96	0.80	0.49
C53-55	Uterus	–	–	–	–	–	–	–	–	618	12.5	0.03	0.43	1.2	0.78	0.69	0.42
C60-63	Male genital organs	75	1.7	0.07	0.03	0.09	0.22	0.08	0.06	–	–	–	–	–	–	–	–
C64-68	Urinary tract	61	1.4	0.05	0.02	0.08	0.17	0.07	0.06	67	1.4	0.05	0.02	0.11	0.14	0.07	0.06
C69-72	Eye, brain, and other parts of the central nervous system	10	0.22	0.04	0.01	0.01	0.01	0.01	0.02	11	0.22	0.03	0.02	0.00	0.01	0.01	0.03
C73-75	Thyroid and other endocrine glands	10	0.22	0.00	0.00	0.01	0.04	0.01	0.01	15	0.30	0.00	0.01	0.02	0.04	0.02	0.01
C76, C77, C80	Other sites, lymph nodes, and unknown primary site	82	1.8	0.04	0.03	0.09	0.29	0.09	0.06	87	1.8	0.02	0.06	0.13	0.17	0.10	0.06

*CR, crude rate; ASR, age-standardized rate*.

For STS, except for the sarcomas NOS, the most common histological subtype was GISS (16.8%), which was followed by fibrosarcoma (7.7%), lipoblastoma (7.5%), leiomyosarcoma (7.3%), and malignant peripheral nerve sheath tumor (MPNST) (5.1%). For bone sarcomas, osteosarcoma (43.0%), giant cell sarcoma (17.4%), and chondrosarcoma (16.1%) were the most three subtypes.

### Mortality

There were 4,279 death cases with sarcomas in Shanghai during 2002–2014, 2,191 (51.2%) for males and 2,088 (48.8%) for females, respectively. The crude annual mortality rate was 2.4/10^5^ and the mortality rate adjusted by the world standard population was 1.3/10^5^. Age-adjusted mortality due to sarcomas was slightly higher in males (1.5/10^5^) than females (1.2/10^5^).

Leiomyosarcoma was the most common subtype, comprising 9.9% of all death cases, followed by lipoblastoma (6.4%) and osteosarcoma (6.3%). About 3.6% of sarcomas death cases occurred in children and adolescents (0–19 years), and osteosarcoma composed of 40.8% in this age group. Majority occurred in the 65+ group with 55.5% of total death cases (Table 4).

**Table 4:** Mortality of sarcomas by age, gender, and histologic type, Shanghai, 2002–2014.

**Histologic group**	**Male**								**Female**							
			**Mortality rates (1/100,000)**								**Mortality rates (1/100,000)**					
	***N***	**%**	**Ages 0–19**	**Ages 20–44**	**Ages 45–64**	**Ages 65+**	**CR**	ASR^*^	***N***	**%**	**Ages 0–19**	**Ages 20–44**	**Ages 45–64**	**Ages 65+**	**CR**	ASR^*^
Sarcoma NOS	888	40.5	0.09	0.17	0.90	4.1	0.99	0.52	867	41.5	0.07	0.21	0.78	3.5	0.96	0.46
Osteosarcoma	153	7.0	0.29	0.12	0.14	0.26	0.17	0.18	117	5.6	0.20	0.10	0.08	0.23	0.13	0.13
Chondrosarcoma	32	1.5	0.01	0.02	0.04	0.09	0.04	0.02	32	1.5	0.00	0.01	0.02	0.13	0.04	0.02
Ewing's sarcoma & PNET	37	1.7	0.06	0.04	0.04	0.02	0.04	0.05	30	1.4	0.06	0.05	0.01	0.03	0.03	0.05
Giant cell sarcoma	20	0.91	0.00	0.02	0.02	0.06	0.02	0.01	16	0.77	0.01	0.02	0.02	0.03	0.02	0.01
Lipoblastoma	157	7.2	0.00	0.03	0.19	0.66	0.17	0.09	118	5.7	0.00	0.02	0.14	0.44	0.13	0.06
Fibrosarcoma	121	5.5	0.02	0.04	0.12	0.53	0.13	0.08	106	5.1	0.01	0.01	0.10	0.46	0.12	0.05
Malignant fibrohistiocytoma	121	5.5	0.01	0.01	0.13	0.60	0.13	0.07	94	4.5	0.00	0.02	0.08	0.40	0.10	0.05
Dermatofibrosarcoma protuberans	22	1.0	0.01	0.01	0.02	0.10	0.02	0.01	15	0.72	0.00	0.00	0.01	0.08	0.02	0.01
Vascular sarcoma	68	3.1	0.00	0.01	0.07	0.33	0.08	0.04	41	2.0	0.01	0.00	0.06	0.13	0.05	0.02
Rhabdosarcoma	56	2.6	0.20	0.02	0.04	0.08	0.06	0.10	45	2.2	0.06	0.04	0.03	0.10	0.05	0.05
Leiomyosarcomas	151	6.9	0.00	0.03	0.14	0.74	0.17	0.09	274	13.1	0.00	0.08	0.40	0.81	0.30	0.15
Gastrointestinal stromal sarcoma	148	6.8	0.00	0.01	0.11	0.84	0.16	0.08	104	5.0	0.00	0.01	0.07	0.52	0.12	0.04
Ameloblastoma	1	0.05	0.00	0.00	0.00	0.01	0.00	0.00	0	0.00	0.00	0.00	0.00	0.00	0.00	0.00
Malignant peripheral nerve sheath tumor (MPNST)	113	5.2	0.02	0.04	0.12	0.48	0.13	0.07	94	4.5	0.03	0.04	0.11	0.28	0.10	0.06
Synovial sarcoma	24	1.1	0.01	0.02	0.04	0.04	0.03	0.02	24	1.2	0.01	0.03	0.04	0.03	0.03	0.02
Stromal sarcoma	13	0.59	0.00	0.00	0.02	0.05	0.01	0.01	64	3.1	0.00	0.01	0.08	0.22	0.07	0.03
Clear cell sarcoma	7	0.32	0.00	0.01	0.01	0.02	0.01	0.00	10	0.48	0.01	0.01	0.01	0.03	0.01	0.01
Myxosarcoma	13	0.59	0.00	0.00	0.01	0.07	0.01	0.01	6	0.29	0.00	0.00	0.01	0.01	0.01	0.00
Malignant mesenchymoma	35	1.6	0.00	0.00	0.05	0.14	0.04	0.02	22	1.1	0.01	0.01	0.03	0.06	0.02	0.01
Embryonic sarcoma	0	0.00	0.00	0.00	0.00	0.00	0.00	0.00	1	0.05	0.01	0.00	0.00	0.00	0.00	0.00
Kaposi's sarcoma	4	0.18	0.00	0.00	0.00	0.02	0.00	0.00	2	0.10	0.00	0.00	0.00	0.01	0.00	0.00
Granulosa cell sarcoma	3	0.14	0.00	0.00	0.00	0.02	0.00	0.00	2	0.10	0.00	0.00	0.01	0.00	0.00	0.00
Alveolar soft part sarcoma (ASPS)	4	0.18	0.01	0.00	0.00	0.02	0.00	0.00	4	0.19	0.00	0.01	0.00	0.00	0.00	0.00
Total	2,191	100.0	0.72	0.60	2.2	9.3	2.4	1.5	2,088	100.0	0.49	0.67	2.1	7.5	2.3	1.2

* *Adjusted by the world standard population. CR, crude rate; ASR, age-standardized rate; NOS, not otherwise specified; PNET, primitive neuroectodermal tumors*.

Among those death cases, 88.1% were located in STS and 11.9% in bone and joint. The CR and ASR of mortality were 2.1/10^5^ and 1.0/10^5^ for STS, 0.3/10^5^ and 0.2/10^5^ for bone sarcomas, respectively. About 39.4% of STS death cases were from connective, subcutaneous, and other soft tissues (ICD-10: C49), followed by digestive organs (24.0%), and retropheritoneum and peritoneum (7.9%) (Table 5).

**Table 5:** Mortality of sarcomas by age, gender, and primary site, Shanghai, 2002–2014.

**ICD-O**	**Primary sites**	**Male**								**Female**							
				**Mortality rates (1/100,000)**								**Mortality rates (1/100,000)**					
		***N***	**%**	**Ages 0–19**	**Ages 20–44**	**Ages 45–64**	**Ages 65+**	**CR**	**ASR**	***N***	**%**	**Ages 0–19**	**Ages 20–44**	**Ages 45–64**	**Ages 65+**	**CR**	**ASR**
C00-14	Lip, oral cavity, and pharynx	24	1.1	0.00	0.01	0.03	0.09	0.03	0.01	6	0.29	0.00	0.01	0.00	0.03	0.01	0.00
C15-26	Digestive organs	501	22.9	0.00	0.07	0.47	2.5	0.56	0.27	405	19.4	0.02	0.06	0.28	1.91	0.45	0.19
C16	Stomach	206	9.4	0.00	0.02	0.15	1.2	0.23	0.11	194	9.3	0.00	0.03	0.09	1.00	0.22	0.08
C30-39	Respiratory system and intrathoracic organs	152	6.9	0.03	0.03	0.20	0.56	0.17	0.09	74	3.5	0.02	0.05	0.12	0.13	0.08	0.05
C40-41	Bone and Joint	286	13.1	0.37	0.21	0.29	0.60	0.32	0.29	225	10.8	0.23	0.16	0.17	0.60	0.25	0.19
C44	Skin	36	1.6	0.01	0.01	0.02	0.21	0.04	0.02	26	1.3	0.00	0.00	0.02	0.13	0.03	0.01
C47	Peripheral nerve and autonomic nerve system	78	3.6	0.00	0.03	0.08	0.32	0.09	0.05	77	3.7	0.05	0.04	0.08	0.21	0.09	0.06
C48	Retropheritoneum and peritoneum	161	7.4	0.00	0.03	0.21	0.65	0.18	0.09	175	8.4	0.00	0.05	0.25	0.53	0.19	0.10
C49	Connective, subcutaneous and other soft tissues	805	36.7	0.21	0.17	0.75	3.8	0.89	0.52	681	32.6	0.10	0.20	0.53	2.83	0.76	0.37
C50	Breast	3	0.14	0.00	0.00	0.00	0.01	0.00	0.00	40	1.9	0.00	0.01	0.06	0.11	0.04	0.02
C51-57	Female genital organs	–	–	–	–	–	–	–	–	290	13.9	0.01	0.06	0.48	0.76	0.32	0.16
C53-55	Uterus	–	–	–	–	–	–	–	–	238	11.4	0.00	0.05	0.42	0.58	0.26	0.13
C60-63	Male genital organs	36	1.6	0.05	0.01	0.03	0.12	0.04	0.03	–	–	–	–	–	–	–	–
C64-68	Urinary tract	43	2.0	0.02	0.01	0.06	0.16	0.05	0.03	30	1.4	0.02	0.00	0.03	0.10	0.03	0.02
C69-72	Eye, brain, and other parts of the central nervous system	9	0.41	0.03	0.01	0.01	0.01	0.01	0.02	9	0.43	0.02	0.02	0.00	0.01	0.01	0.02
C73-75	Thyroid and other endocrine glands	6	0.27	0.00	0.00	0.01	0.02	0.01	0.00	10	0.48	0.00	0.00	0.01	0.04	0.01	0.01
C76, C77, C80	Other sites, lymph nodes and unknown primary site	51	2.3	0.01	0.01	0.04	0.26	0.06	0.03	40	1.9	0.02	0.01	0.05	0.12	0.04	0.02

*CR, crude rate; ASR, age-standardized rate*.

### Trends

**Figure 1:**
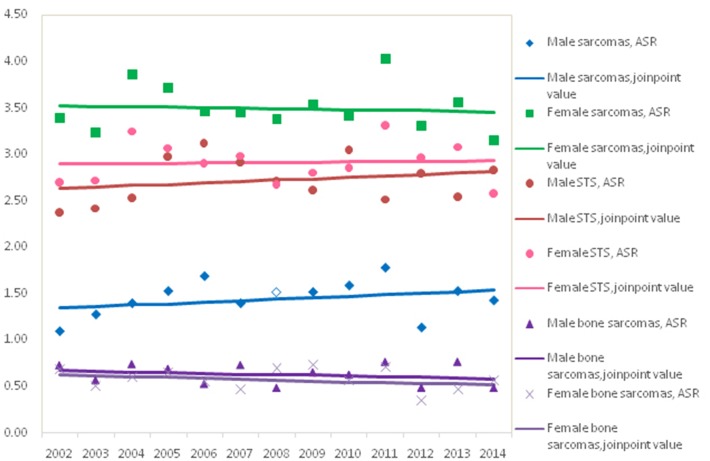
Trends for age-adjusted incidence of sarcomas by gender, Shangai, 2012–2014.

Figure 1 showed the trends in the incidence for all sarcomas combined, STS and bone sarcomas during 2002–2014. No significant incidence trend for all sarcomas combined was observed in males and females, with an APC of 0.3% (95% CI: −0.9, 1.4%) and −0.2% (95% CI: −1.3, 1.0%) in ASRs by Joinpoint regression, respectively. The trends of incidence rates for STS and bone sarcomas were not significant for both genders. Further analysis showed that the trends of top five subgroups showed that incidence rates continued to decline during 2002–2014 for leiomyosarcomas, fibrosarcoma, and MPNST, while the ASR of lipoblastoma stabilized and GISS increased significantly (data not shown).

**Figure 2:**
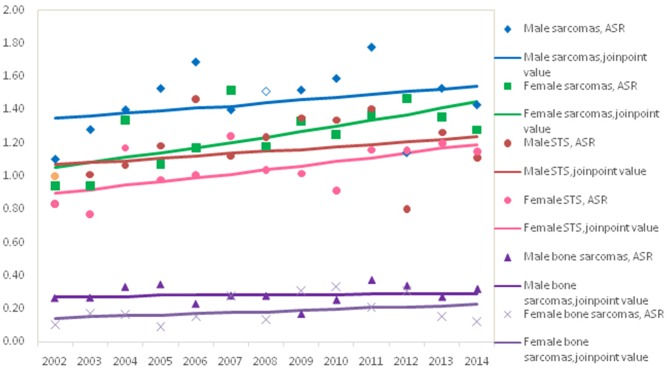
Trends for age-adjusted mortality of sarcomas by gender, Shangai, 2012–2014.

The trend in the mortality of total sarcomas during 2002–2014 increased significantly with APC 2.7% (95% CI: 0.7, 4.7%) for females, while it was not significant for males with APC 1.1% (95% CI: −1.1, 3.5%). Further analysis found that the significant rising trend only existed in the ASRs for female STS with APC 2.3% (95% CI: 0.3, 4.4%), but not for female bone sarcomas during the entire time period, as shown in Figure 2. In female, the morality rates increased but not significantly for lipoblastoma (APC 5.7, 95% CI: −0.2, 11.1%).

## Discussion

The results presented in this paper gave for the first time a comprehensive analysis focusing the incidence and mortality of sarcomas in Shanghai. The recent 2002 WHO criteria were used to recategorize histologic subtypes of sarcomas in the present study to facilitate comparison with other studies. Total 9,440 cases were diagnosed during 2002–2014 in SCR, of which 12.1% were bone sarcomas and 87.9% were soft tissue sarcomas. The annual ASR for all sarcomas combined was 3.4/10^5^, and the ASRs for STS and bone sarcomas were 2.8/10^5^ and 0.6/10^5^, respectively.

Sarcomas account for about 1% of all malignant tumors and the total incidence ~2–4 per 100,000 population (1, 2). Data from *CI5* showed that sarcoma incidence rates were comparable throughout much of the world (16). Previous studies that examined the incidence of sarcomas combined with STS and bone sarcomas were limited and there were marked variations in the distribution of subtypes. Population-based data on incidence of sarcomas in Europe was investigated in a study from RARECARE project (4), covering 27,908 incident cases diagnosed during 1995–2002 in the EU27 countries with crude incidence of 5.6/10^5^, of which 84% were soft tissue sarcomas and 14% were bone sarcomas, similar to our findings.

The ASR of STS in Shanghai (2.8/10^5^) were comparable with the report from Austrian National Cancer Registry (ASR 2.4/10^5^) (17) and were lower than the findings from SEER program in the USA (total 5.0/10^5^ with US 2000 standard population) (18) and from RARECARE project in Europe (total ASR 4.2/10^5^) (4). A previous report in Beijing on STS, only including the cases diagnosed with sites of C47 and C49, showed that the incidence was lower than our findings, with CR and ASR 1.2/10^5^ and 0.9/10^5^, respectively (19). Bone sarcomas were relatively rare and the ASR (0.6/10^5^) in Shanghai was similar to the finding from RARECARE project in Europe (ASR 0.8/10^5^) (4) and a report in Taiwan (ASR 0.67/10^5^) (7).

The histological and molecular classification of sarcomas has been revised with the progress of new techniques, such as immunohistochemistry, multiplex PCR and sequencing, which would be reflected by the distribution and trends of histological subgroups. In this study, the three most frequent histological subtypes among STS were GISS, fibrosarcoma, and lipoblastoma, respectively. RARECARE project showed that leiomyosarcoma was the most frequent type, whereas others showed that the most common histology was liposarcoma (4). There has been a significant steadily increasing numbers of GISS in Shanghai since 2010 accounting for about range of 14.8–18.5% of all sarcomas. GISS were registered with six cases only in 2002 and varied a range of 0.4–5.9% before 2010. One possible reason was that GISS had been diagnosed as sarcomas NOS. The total proportion of sarcomas NOS in this study was 31.2% during the entire period and the percent declined significantly since 2010, accounting for about 20%. The “*Chinese consensus guidelines for diagnosis and management of gastrointestinal stromal tumor*” was published in 2008 and then it has received more attention with several revised edition. The application of techniques of immunohistochemistry and molecular pathology resulted in more GISS identification. Another possible explanation was that the incidence rates of leimyosarcomas decreased significantly during this period. It was reported that before using immunohistochemistry, some GISS might had been identified as leiomyosarcoma (20).

Age is an important determinant of sarcoma occurrence. Incidence of STS increases more dramatically after 50 years old. Generally, malignant bone tumors have a stable incidence rate across all ages. However, in adolescents and young adults, there is a noticeable increase (1). Similar age patterns were found in this study. A considerable variation in incidence patterns of sarcomas by histologic subtypes in this study was observed, which supported the notion that these tumors are etiologically distinct and should be considered separately in studies of potential risk factors, in accord with previous epidemiologic studies (18).

During 2002–2014, the trends in the ASRs of incidence for all sarcomas combined, STS and bone sarcomas were not significant for males and females. An upward trend in the incidence of STS overall and for females was seen in Osaka, Japan during 1978–2007 (21). However, a population-based epidemiologic study in Austria for the period 1984–2004 (17) has not confirmed the increasing incidence rates of STS. It mentioned that different inclusion criteria (such as Kaposi's sarcoma and dermatofibrosarcoma) and classifications in the various studies would explain the increase of incidence in some studies rather than true increase of STS due to new or accumulated risk factors (17).

The causes of most sarcomas are unknown. Both genetic and environmental factors likely contribute to the etiology of sarcomas (1, 8, 21). The rarity of the disease combined with the diverse number of subtypes make sarcomas difficulty to study and the epidemiology and etiology of sarcomas are not well-understood. Environmental factors that increase sarcoma risk include radiation exposure and chemical carcinogens (8). Ionizing radiation exposure, especially by means of radiotherapy for a previous cancer, has been shown to be strongly associated with secondary sarcoma development (1, 22). There was an increasing incidence of second sarcomas among cancer survivors, and one may speculate a relation to the intensified use of cytotoxic treatment of the preceding malignancy (23). Other risk factors include occupational exposure to certain chemicals, including herbicides such as phenoxyacetic acids. HIV-positive individuals have an increased risk for Kaposi's sarcoma. Several familial cancer syndromes confer sarcoma pre-disposition, such as the LFS (8). No study has been implemented in Shanghai about the risk factors of sarcomas and these data serve to illustrate the complexity of sarcomas.

To our knowledge, no population-based study to date has evaluated the mortality of sarcomas for all types combined and the histological subtypes. In this study, the ASR for mortality of sarcomas combined was 1.3/10^5^ and the ASRs of mortality for STS and bone sarcomas were 1.0/10^5^ and 0.2/10^5^, respectively. For STS, except for the sarcomas NOS, leiomyosarcomas was the most common subtype among death cases of sarcomas and majority occurred in the old over 65+ years. For bone sarcomas, about 40% of cases occurred in children and adolescents (0–19 years).

No substantial changes were found in the mortality rates of sarcomas combined and bone sarcomas for males and females. However, a modest significant increase in average annual mortality rates (APC, 2.3%; 95% CI: 0.3, 4.4%) was observed for STS among females, not males. The mortality increasing of female STS was maybe due to in part to the distribution change of subtypes and the poor survival of lipoblastoma in females. One should be noticed that some of sarcoma subgroups were very rare and varied a lot with a wide range of 95% CI. A study from a medical unit in UK showed that there had been no significant change in 1 year mortality rate of STS during 1985–2010, and TNM stage was a useful predictor (24). Survival studies on sarcomas and its subgroups are warranted.

This study included the sarcomas at all sites including skin and visceral sarcomas, and not only bone and soft tissue tumors. SCR was a population-based registry and has been a regular contributor to the *CI5*. The quality of data in this report was high with 94.4% of MV. Otherwise, there were several potential limitations in our study. The use of new techniques may be systematically under- or over-represented, which influenced the patterns and the trends. There was evidence that the *gold standard* pathologic diagnosis was not consistently reliable for sarcomas and the chances to misclassify the histology for a pathologist also existed (8, 25, 26) Although this study focused on the data after 2002 and some advent of ancillary technologies, such as immunohistochemistry and molecular genetics/molecular cytogenetics, had been applied in Shanghai, there were over 30% of sarcomas NOS in this study. It's a good point that the proportion of sarcomas NOS reduced to about 20% after 2010. In addition, as previously described, some of the sarcoma subtypes are uncommon and generalizations concerning incidence and mortality rates are difficult to make.

The diversity and rarity of sarcomas suggested that a cooperative networking in prevention, diagnosis, therapy, and research for this rare type of cancers was warranted. With the ongoing collection of the sarcoma cases, accumulative detailed information may reveal more subtle etiologic clues and provide more evidence for decision on effective therapy.

## Ethics Statement

The study was approved and the need for consent was waived by the institutional review board (IRB) of Shanghai Municipal Center for Disease Control and Prevention. In this study, only data in annual cancer report was used and no information to identify individual subjects was included.

## Author Contributions

FC, YY, BP, ZY, and LW contributed conception and design of the study. WChunx, WChunf, and BP collected and organized the database. BP and WChunx performed the statistical analysis. BP and ZY draft the manuscript. SY, ZC, XJ, LJ, KL, CZ, and ZW revised and made the decision to submit for publication. All authors contributed to manuscript revision, read, and approved the submitted version.

### Conflict of Interest Statement

The authors declare that the research was conducted in the absence of any commercial or financial relationships that could be construed as a potential conflict of interest.
